# Primary Care Nurses’ Experiences and Strategies for Addressing Communication Barriers in Caring for Migrant Patients: A Qualitative Study

**DOI:** 10.3390/healthcare14213774

**Published:** 2026-11-04

**Authors:** María Muñoz Montoya, María del Mar Torres López, María Mar López-Rodríguez, Alda Elena Cortés-Rodríguez, José Granero-Molina, María del Mar Jiménez-Lasserrotte

**Affiliations:** 1 Distrito Poniente, Servicio Andaluz de Salud, 04700 El Ejido, Almería, Spain; mariamumon@gmail.com; 2 Hospital Universitario de Poniente, Servicio Andaluz de Salud, 04700 El Ejido, Almería, Spain; mtl112@inlumine.ual.es; 3 Department of Nursing, Physiotherapy and Medicine, University of Almería, 04120 Almería, Spain; cra566@ual.es (A.E.C.-R.); jgranero@ual.es (J.G.-M.); mjl095@ual.es (M.d.M.J.-L.)

**Keywords:** primary health care, language barriers, nursing, health equity, migrants, qualitative study

## Abstract

**Highlights:**

**What are the main findings?**

- Primary care nurses perceive language barriers as factors disrupting nurse–patient communication and continuity of care while increasing emotional strain and workload.
- Nurses perceive gender and cultural factors as influences on language-related difficulties, relying on improvised communication strategies, digital translation tools, and informal community support in the absence of formal resources.

**What are the implications of the main findings?**

- Primary care services need structured language-support pathways, including professional interpreting and culturally adapted communication resources, to promote safer and more equitable care.
- Addressing language barriers requires an organizational response combining standardized protocols, intercultural training, and institutional support, rather than relying on individual adaptation by nurses.

**Abstract:**

Communication barriers are an increasingly important issue in primary care due to the growing migrant population. In Andalusia, particularly in Almería, this situation poses a significant challenge for nurses working in primary care settings. Linguistic diversity can affect the nurse–patient relationship, exchange of health information and continuity of care. Objectives: The aim of this study was to explore primary care nurses’ experiences and strategies for addressing communication barriers when caring for migrant patients. Methods: A descriptive qualitative study was conducted. Twenty-two nurses were recruited through convenience sampling and interviewed. Results: Data were analyzed using thematic analysis, and three main themes emerged: (1) language barriers, discontinuity of care, and nursing workload; (2) nurses’ perceptions of gender and cultural influences on communication; and (3) adaptive responses, community support, and governance demands. Conclusions: Communication barriers in primary care, particularly those related to language, were perceived by nurses as affecting the quality and continuity of care and contributing to emotional distress. In the absence of institutional resources, nurses rely on improvised strategies. The findings highlight the need for institutional responses that promote equitable, culturally competent, and patient-centered care.

## 1. Introduction

International migration has contributed to increasingly diverse primary care populations, bringing patients and health professionals with different linguistic, cultural, and social backgrounds into everyday contact [1,2]. In Spain, this diversity is particularly visible in regions with sustained migrant settlement, including Andalusia, where primary care services are provided to populations with heterogeneous origins and health needs [3,4]. The Spanish migration context also encompasses different migration routes and administrative situations, including irregular arrivals by sea [5,6], and irregular status may create additional barriers to health care access and challenge the principles of universal health coverage (UHC) promoted by the World Health Organization (WHO) [7,8]. Nevertheless, administrative status represents only one dimension of a much broader and more diverse reality. Across these different circumstances, the ability of patients and professionals to communicate, understand one another, and establish a therapeutic relationship remains fundamental to the delivery of equitable and person-centered care.

Communication can become particularly complex when patients and health professionals do not share a common language. Language discordance may hinder the collection of an accurate clinical history, the explanation of diagnoses and treatments, the assessment of patients’ understanding, and their informed participation in decisions about care [9,10,11]. Difficulties in understanding or being understood may also generate frustration and undermine trust in health services [10]. However, communication barriers are not exclusively linguistic. Differences in health literacy, beliefs, expectations, and cultural understandings of health and illness can influence how symptoms are described, how professional recommendations are interpreted, and how care is negotiated within the clinical encounter [11,12,13]. Communication with migrant patients therefore involves both the accurate exchange of information and the ability to build a shared understanding within a culturally and linguistically diverse context.

These issues are particularly relevant in primary care nursing, where communication is not an isolated component of care but a central part of everyday practice. The nurse–patient relationship is grounded in therapeutic communication [14], and nurses depend on dialogue to assess health needs, provide preventive care and health education, explain medication and treatment plans, support adherence, and ensure continuity of follow-up. When communication is limited, even routine activities may require additional time and effort, while uncertainty may remain about whether information has been understood or whether recommendations can be followed safely [9,15]. Repeated encounters under these conditions may also increase the practical and emotional demands placed on nurses. Similar difficulties have been described in other health care settings [16], but they acquire particular significance in primary care because of its emphasis on accessibility, prevention, continuity, and sustained relationships with patients and families.

In the absence of a shared language, health professionals may resort to different strategies to make communication possible. Digital translation applications, gestures and other non-verbal resources, relatives or informal mediators, and professional interpreting services are among the options described in clinical practice [17,18]. However, these strategies are not equivalent in terms of accuracy, confidentiality, availability, or suitability for complex clinical information. Professional interpreting may improve communication and the quality of care [18], whereas improvised solutions can be insufficient when precise information must be exchanged [17]. Therefore, the way communication barriers are managed depends not only on the skills and initiative of individual nurses but also on the resources available within health services, the organization of care, and the extent to which professionals are supported in providing culturally responsive care [12,13].

Although language barriers and communication support in health care have been widely examined [9,15,17,18], understanding how these challenges are experienced in the everyday work of primary care nurses remains important. Nurses repeatedly encounter patients across preventive care, health education, treatment follow-up, vaccination, chronic disease management, and other routine consultations, making them particularly well placed to identify where communication breaks down and how it is addressed in practice. Examining their experiences can therefore provide insight into not only the barriers they perceive but also the strategies they use, the limitations of improvised responses, and the support they consider necessary. This perspective may help identify areas in which individual practice and organizational measures can be strengthened to promote safer, more equitable, and culturally responsive primary care. Therefore, the aim of this study was to explore primary care nurses’ experiences and strategies for addressing communication barriers when caring for migrant patients.

## 2. Materials and Methods

### 2.1. Design

A descriptive qualitative study was conducted. This methodological design enables a comprehensive description of a topic based on participants’ accounts and perspectives [19]. This approach is appropriate for exploring experiences in health care settings from the perspective of health professionals [20]. The study was reported in accordance with the Consolidated Criteria for Reporting Qualitative Research (COREQ) [21].

### 2.2. Participants and Setting

This study was conducted at eight primary care centers and local clinics in the province of Almería belonging to the Poniente Health District and the Almería Health District. To include primary care settings with varied geographical characteristics, urban, semi-urban coastal, and rural centers located in areas of intense agricultural activity were selected. The proportion of registered migrants at these centers ranges from 20% to 45%, depending on the area. The primary care centers were intentionally selected according to their geographical characteristics and the proportion of patients of migrant origin attending them. Nurses working at these centers were recruited through convenience sampling, based on their availability and willingness to participate voluntarily. The inclusion criteria were (1) being a primary care nurse; (2) having at least one year of experience caring for migrant patients; and (3) having provided informed consent. Health district coordinators facilitated access to the selected centers and invited nurses to participate voluntarily. A preliminary meeting was held with key informants (district coordinators). They helped select the primary care centers with the highest proportion of patients with migrant origin, contacted the nurses working at those centers, explained the study protocol to them, and invited them to participate voluntarily. The researchers contacted the participants by email or phone, scheduling interviews based on their availability. The district coordinators were not present during the consent process or during the interviews. Twenty-five nurses were invited to participate, of whom three declined due to scheduling constraints. The final sample comprised 22 nurses working at primary care centers. Participants’ sociodemographic characteristics are presented in Table 1.

### 2.3. Data Collection

Data were collected through 22 in-depth interviews (IDIs) conducted between February and May 2026. Interviews took place in the nursing consultation room at each participant’s health care center. Access to potential participants was facilitated by primary care area coordinators. Each participant completed a single IDI, with a mean duration of 57 min, attended only by the participant and one researcher. The IDIs were conducted by two researchers with training in qualitative research and migration (M.d.M.J.-L., M.d.M.T.L.), using a semi-structured interview guide that had been previously piloted (Table 2). This interview guide was developed based on a literature review of the topic. The interview protocol was pilot-tested with two primary care nurses who had five years of experience working in settings that serve migrant populations. Following the pilot test, minor wording changes were made to two questions to improve clarity; no changes to the original script were necessary. Before each interview, the researchers introduced themselves and explained the purpose of the study. The researchers then collected the participants’ sociodemographic data, assured confidentiality, and obtained written informed consent. All interviews were conducted in Spanish and audio-recorded with participants’ permission. Transcription and analysis were carried out in Spanish. Quotations presented in the manuscript were translated into English by a bilingual native British English speaker residing in Spain and reviewed by a bilingual Spanish author. By the 20th interview, the research team considered that the information collected was sufficient to address the study objective. Two additional interviews were conducted to further assess this judgement. Field notes of participants’ non-verbal cues and researchers’ thoughts were written down in a reflective journal during the interviews.

### 2.4. Data Analysis

All IDIs were transcribed and, together with the field notes, imported into an ATLAS.ti 24 project for analysis. The researchers created notes in ATLAS.ti 24 in which they recorded their reflections, pre-analytical ideas, and interpretations of the data to support their analytical work. The initial analysis of all transcripts was conducted independently by two researchers (M.d.M.T.L. and M.M.M.) and subsequently cross-checked collaboratively. Through reflective dialogue and regular meetings with the rest of the research team (M.d.M.J.-L. and M.d.M.L.-R.), the initial codes were compared and interpretations were discussed. The final thematic structure was developed through reflective discussions within the research team, during which interpretations were examined and themes were refined. Data were analyzed following the six-phase thematic analysis approach described by Braun and Clarke [22]: (1) familiarization with the data: all transcripts were read in full to gain an overall understanding of participants’ accounts and were reread to identify and record initial ideas; (2) systematic coding of the data: transcripts were systematically coded by identifying meaningful segments of text and assigning codes to capture their content. Open and in vivo coding were used where appropriate, supported by corresponding functions in ATLAS.ti; (3) generating initial themes from the codes: codes sharing a common meaning and linked by a central organizing concept were grouped to develop candidate themes. A table with examples of the coding strategy using significant quotes, subthemes, and topics was created (Table 3); (4) developing and reviewing themes: the candidate themes were reviewed to assess their coherence with the coded data and the extracts supporting each theme; (5) refining, defining, and naming themes: themes were refined, clearly defined, and assigned names that reflected their central meaning and scope; and (6) writing the report: representative quotations were selected, and synthesized descriptions of each theme and subtheme were developed. The findings were interpreted in relation to the research question and discussed in light of relevant scientific literature.

### 2.5. Rigor and Reflexivity

Methodological rigor was addressed using the quality criteria proposed by Lincoln and Guba [23]. Credibility was achieved by thoroughly documenting data collection and through triangulation among researchers during the coding process. Regular peer review sessions were held to discuss the coding schemes, which were reviewed by researchers with expertise in qualitative research and migration. Transferability was supported by a detailed description of the primary care clinical context, the intentional selection of healthcare settings and convenience sampling of participating nurses, and the researchers’ reflexivity. Dependability was addressed by maintaining a detailed record of the study process, including the procedures used for data collection and analysis (transcripts, field notes) and the methodological decisions made throughout the research process. Confirmability was supported by the use of verbatim quotes from the interviews to link the findings to the participants’ narratives. Several researchers verified that the data (participants’ quotes) were consistent with the codes, themes, and subthemes. No verification was conducted by the participants due to the nurses’ heavy clinical workload. Reflexivity: The researchers who conducted the interviews (M.d.M.J.-L., Ph.D., and M.d.M.T.-L., a clinical nurse with the Andalusian Health Service) are qualitative researchers in the field of health with experience in nursing and transcultural care. The initial coding of all transcripts was carried out independently by two clinical nurses at the Andalusian Health Service (M.d.M.T.-L. and M.M.-M.) and was subsequently cross-checked collaboratively. Through iterative reflective dialogue and periodic sessions with the senior researchers affiliated with the university (M.d.M.J.-L., Ph.D., and M.d.M.L.-R., Ph.D.), the initial codes were compared and the interpretations were critically discussed. None of the interviewers had a pre-existing hierarchical relationship or direct supervisory role with the study participants. To minimize internal bias and prevent clinical assumptions from influencing the interpretation, the researchers maintained reflective journals throughout the data collection and coding iterations. The multidisciplinary background of the research team—which combined primary and hospital nursing, bioethics and the humanities, and public health—provided complementary perspectives during the interpretation process. This reflective approach enriched the thematic analysis and strengthened the credibility of the findings.

### 2.6. Ethical Considerations

This study was conducted following the ethical principles of the Declaration of Helsinki for biomedical research involving human participants. Participants were informed about the purpose of the study and the voluntary nature of their participation. Written informed consent was obtained before participation, including consent to audio-record the interviews. Participation was requested directly by the researchers in private consultation rooms, ensuring that the nurses could freely decide whether to participate or decline without any professional or hierarchical pressure. Participants were informed of their right to withdraw from the study at any time. Confidentiality and data protection were ensured in accordance with Spanish Organic Law 3/2018 of 5 December on the Protection of Personal Data and Guarantee of Digital Rights and Regulation (EU) 2016/679 of the European Parliament and of the Council of 27 April 2016. The principal investigator stored the research data securely, and each participant was assigned a unique code to protect their identity throughout the research process. The study received ethical approval from the Research Ethics Committee of the Department of Physiotherapy, Nursing and Medicine at the University of Almería (EFM-410.25).

## 3. Results

**Figure 1:**
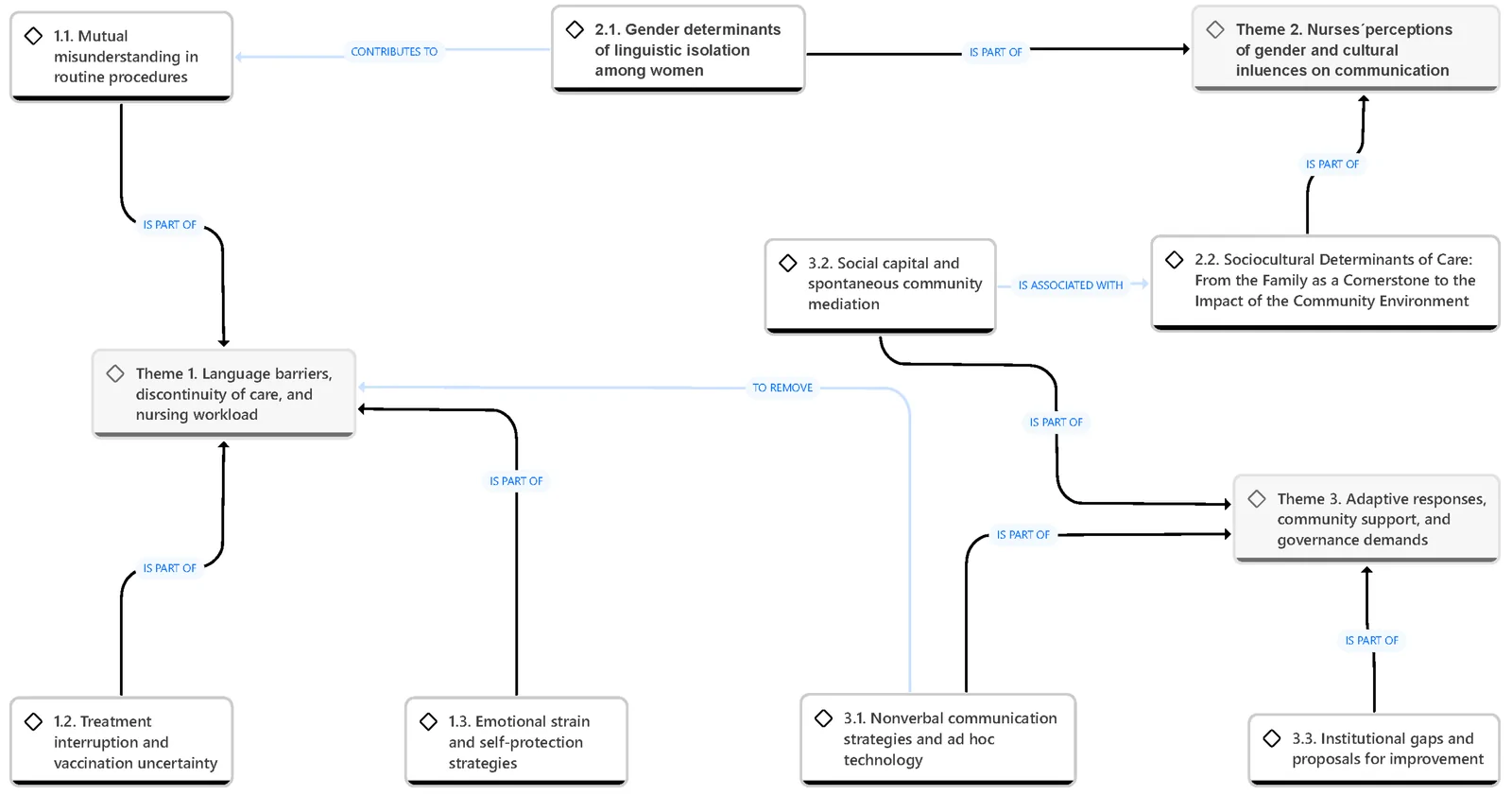
Conceptual map of themes and subthemes.

The sample comprised 22 primary care nurses, of whom 4 (18%) were men and 18 (82%) were women. The mean age was 42.4 years, and the mean professional experience was 15.4 years. Thematic analysis identified three main themes and eight subthemes. These findings describe nurses’ experiences and strategies for addressing communication barriers when caring for migrant patients (Table 4). The conceptual and hierarchical relationships among these categories are illustrated in the thematic map (Figure 1).

### 3.1. Theme 1. Language Barriers, Discontinuity of Care, and Nursing Workload

Communication difficulties between nurses and migrant patients who did not speak Spanish affected clinical interactions. These difficulties compromised information exchange and the quality of the therapeutic relationship in everyday practice, while requiring nurses to rely on improvised strategies that complicated the flow of consultations and clinical follow-up and generated substantial emotional strain.

#### 3.1.1. Mutual Misunderstanding in Routine Procedures

Participants described difficulties maintaining fluent communication with migrant patients, which led to recurrent misunderstandings and the need to repeatedly explain therapeutic instructions. These situations generated persistent feelings of insecurity and frustration among nurses.

*“Many times, you are talking to them and they keep saying yes, yes, but in reality they do not understand what you are telling them”*

*(IDI-7).*

Some participants noted that these difficulties became more pronounced in clinically sensitive situations, such as symptom assessment, explanation of treatment regimens, or shared decision-making. In such situations, mutual misunderstanding complicates clinical decision-making and raises concerns about patient safety, particularly regarding medication administration.

*“There are many times when you do not know whether the person has understood you… and with important things, such as medication, it is frightening”*

*(IDI-5).*

Some nurses noted that even routine procedures, such as scheduling appointments or explaining administration instructions, became difficult in the absence of shared language to ensure mutual understanding.

*“You try to tell them to come back another day or in two days and they say yes, but then you do not know whether they understood, and many times they do not come back”*

*(IDI-6).*

#### 3.1.2. Treatment Interruption and Vaccination Uncertainty

Nurses described these communication difficulties as affecting treatment continuity and clinical follow-up, in some cases contributing to treatment discontinuation when patients did not understand the need to return for follow-up. As one participant said:

*“They stop coming, and when you ask them, they tell you they did not understand what they had to do or […] that they had to come back”*

*(IDI-3).*

Under these circumstances, nursing staff described difficulties verifying whether treatment instructions had been followed correctly and monitoring changes in the patient’s clinical status, creating uncertainty about treatment adherence.

*“When the language is not clear, follow-up is very difficult. You ask them […] and many times they cannot explain it to you”*

*(IDI-4).*

*“There are treatments they stop because they do not understand what they are for or how to take them”*

*(IDI-5).*

One participant identified childhood vaccination as an area of particular complexity, describing difficulty verifying the vaccination status of some patients from Morocco because of perceived differences between vaccination schedules and, in some cases, the absence of official vaccination records.

*“[…] they come from Morocco […] but they do not have documents […], the schedule they have there is different. You do not know whether to revaccinate or wait”*

*(IDI-7).*

#### 3.1.3. Emotional Strain and Self-Protection Strategies

Nurses described the communication gap as an additional source of emotional strain during consultations, expressed through frustration, exhaustion, and fear of making mistakes. These feelings were particularly pronounced when communication difficulties were perceived to affect the quality of care provided. Even after lengthy attempts to explain dosage and care instructions, uncertainty persisted about whether information was correctly understood, which researchers interpreted as a subtle fear of malpractice and professional liability.

*“You make an effort to explain everything and to do your job properly, but when you do not know whether they have understood you… you leave with the feeling that you are not doing it well”*

*(IDI-20).*

The persistence of these experiences contributed to emotional overload and psychological exhaustion among nurses interviewed, who described having to make additional efforts to achieve basic mutual understanding in the absence of institutional support or adequate training.

*“It is very draining […] you end up mentally exhausted, as if you had done twice the work”*

*(IDI-14).*

Considering this persistent unease, one participant reflected on the importance of awareness of personal perceptions and emotional responses when providing care across cultural differences.

*“[…] to provide care in a way that also makes me feel comfortable […] and to change my emotions so that I can deal with different situations”*

*(IDI-15).*

In other cases, this distress influenced nurses’ preferences regarding their work setting. One nurse described preferring, when possible, not to work in centers serving a high proportion of patients who did not speak Spanish as a form of professional self-protection from the daily strain of communication difficulties.

*“I already know which centers have more foreign patients and, if I can choose, I prefer not to go […] because it is very hard not being able to communicate day after day”*

*(IDI-6).*

### 3.2. Theme 2. Nurses’ Perceptions of Gender and Cultural Influences on Communication

Participants perceived that language-related communication difficulties did not affect all migrant patients uniformly. In their accounts, gender-related factors and culturally specific models of care were described as shaping opportunities for linguistic interaction and communication with the health care system.

#### 3.2.1. Gender Determinants of Linguistic Isolation Among Women

Some participants perceived that certain migrant women had fewer opportunities for social interaction outside the home. Nurses interpreted this as potentially limiting opportunities to use and develop Spanish and, in some encounters, as contributing to reliance on male relatives or other third parties during health care interactions.

*“For example, when they come with their wives, we speak to the women, but the men are the ones who answer us…”*

*(IDI-5).*

In participants’ accounts, this perceived reduction in contact with the wider social environment was associated with family and cultural patterns that nurses interpreted as potentially reinforcing social and linguistic isolation. These interpretations reflected nurses’ perspectives on the women they encountered in practice rather than direct accounts from migrant women themselves.

*“They are always at home; they do not go anywhere alone, not even to the health center. That means they hardly hear Spanish and everything depends on the husband”*

*(IDI-13).*

Participants also perceived limited contact with community and social environments as reducing opportunities to use Spanish in everyday interactions, which they associated with difficulties in developing language skills. As one participant noted:

*“[…] they do not learn the language well. They cannot communicate properly because they are not as integrated”*

*(IDI-17).*

From the nurses’ perspective, these circumstances could contribute to communication inequalities in some encounters, particularly when women relied on third parties during health care interactions. Participants perceived that such reliance could limit direct communication with health professionals and the independent exchange of health-related information.

#### 3.2.2. Sociocultural Determinants of Care: From the Family as a Cornerstone to the Impact of the Community Environment

Some nurses highlighted the importance of family caregiving among the migrant families they encountered, particularly in relation to older relatives. They described a preference for home-based care, with family members assuming a central caregiving role. Although these accounts did not directly demonstrate communication barriers, nurses perceived family involvement as relevant to healthcare interactions, particularly when relatives helped patients overcome language difficulties.

*“And they don’t even consider putting their grandfather in a nursing home, even if they could afford it; they just don’t think about it. The grandfather or grandmother has to stay at home because they are the pillar of their family, of their community…”*

*(IDI-11).*

Nurses also perceived differences between health centers in patterns of social interaction and familiarity with local healthcare practices. These observations reflect their experiences in particular settings and do not establish a causal relationship between population diversity and communication.

*“There, [patients from the same background] come together and [researchers’ interpretation: endogamous dynamics] are reinforced, whereas here they are more subdued because there are people from all over. The difference is very noticeable between health centers where almost the entire population is immigrant and those where there is greater diversity”*

*(IDI-12).*

### 3.3. Theme 3. Adaptive Responses, Community Support, and Governance Demands

In the absence of official resources, nurses relied on improvised strategies to bridge language barriers, including visual communication, ad hoc technology, and support from patients’ social networks. However, these informal solutions were perceived as insufficient to address communication barriers systematically, leading nurses to call for structural and organizational responses from the health care system.

#### 3.3.1. Nonverbal Communication Strategies and Ad Hoc Technology

Nurses described using pictographic charts and gestures to identify symptoms or provide simple instructions, although these resources depended largely on individual initiative. These strategies had clear limitations, as they do not allow nurses to explore symptoms in depth or assess whether patients had fully understood the information.

*“I used some sheets with drawings of the human body […] and that was the only thing that worked a little”*

*(IDI-9).*

*“In the end you resort to gestures: pointing to the stomach, pretending to swallow a pill… and hoping they understand you”*

*(IDI-4).*

Nurses also used Google Translate to facilitate simple exchanges, while highlighting its limitations and the risk of misunderstandings when the digital translation software generated inaccurate or nonsensical phrases during clinical interactions. The researchers classified these discrepancies in machine translation as factors that increase the risk of clinical errors and compromise patient safety.

*“Google Translate sometimes helps, but sometimes it says things that have nothing to do with what you mean, and that leaves you even more confused”*

*(IDI-10).*

Professionals also described creating improvised graphic materials during consultations to explain procedures or symptoms when other means of communication were unavailable.

*“I have had to draw things on a piece of paper to explain what was happening because there was no other way for them to understand”*

*(IDI-19).*

Although these strategies helped nurses manage communication in specific situations, their recurrent use reflected the lack of systematic institutional resources to address the linguistic diversity of the population receiving care.

#### 3.3.2. Social Capital and Spontaneous Community Mediation

Participants described informal social networks as a source of communication support. In one account, a community religious leader was described as accompanying patients and helping with translation.

*“The person who runs the mosque helps them; he even gives them Spanish lessons or translates for them if they need it. Many times, he comes with them to the consultation”*

*(IDI-21).*

Patients were also frequently accompanied by friends, and other patients in the waiting room sometimes volunteered to translate. Nurses described this informal support as helping facilitate consultations and reducing the stress associated with language barriers.

*“They come with a friend who speaks a little more Spanish and acts as an interpreter. It is not ideal, but without that we would hardly be able to do anything”*

*(IDI-16).*

*“Sometimes another patient* notices *[…] and offers to help. […] that has been a relief”*

*(IDI-1).*

#### 3.3.3. Institutional Gaps and Proposals for Improvement

Despite the usefulness of these informal networks, nurses described important institutional gaps, including limited access to guidelines, official protocols, and formal training in cultural diversity. These gaps contributed to insecurity in daily practice and increased reliance on informal strategies.

*“We have nothing official for this. No guidelines, no protocols, no materials. Each of us manages as best we can, depending on the situation”*

*(IDI-1).*

*“We have not been trained for this. […] You do everything by intuition”*

*(IDI-18).*

One participant recalled a telephone interpreting service used during the COVID-19 pandemic and perceived its subsequent withdrawal as a loss of formal communication support.

*“During the pandemic they did provide telephone interpreters, but now there is nothing. It was temporary and then it disappeared”*

*(IDI-4).*

In response, nurses proposed improvements, including unified protocols and standardized informational leaflets with multilingual pictograms. They considered that these resources could help standardize the response to language barriers and facilitate the communication of basic health information.

*“It would be good to have something established, to know how to act when you cannot communicate because right now it depends on each person and on the day”*

*(IDI-8).*

*“If we had leaflets in their language or pictures explaining basic things, that alone would be a huge help”*

*(IDI-3).*

One participant proposed real-time translation technology as a potential means of facilitating communication during clinical encounters.

*“They should invent something like earphones that translate what we say […] in real time. That really would be useful”*

*(IDI-14).*

One participant explicitly called for greater health-system responsibility for addressing communication barriers, moving beyond reliance on individual and improvised responses.

*“This needs to be taken seriously from the top […]. It must be something that is built into the system”*

*(IDI-2).*

## 4. Discussion

The aim of this study was to explore primary care nurses’ experiences and strategies for addressing communication barriers when caring for migrant patients. In our study, participants consistently described misunderstandings related to language differences and emphasized the difficulty of determining whether patients had fully understood the information provided. This lack of feedback was perceived as an obstacle to the flow of the consultation and to effective communication. These findings agree with previous research describing communication challenges in nurse–migrant patient encounters [24]. From a broader health-equity perspective, the right to health requires health services to be accessible and non-discriminatory [25], while culturally and linguistically diverse populations may encounter structural and communication-related barriers to health care access [26]. Previous research has also emphasized the importance of communication strategies in language-discordant consultations [27] and associated care in a patient’s preferred or native language with stronger therapeutic relationships and patient participation [28]. Consistent with this literature, nurses in our study perceived language barriers as particularly challenging for therapeutic follow-up and ongoing health assessments because they could not always confirm whether patients had understood the information and recommendations provided. Participants also described linguistic and cultural differences as complicating clinical assessment and reducing the perceived effectiveness of health education. Previous research on cultural competence in primary health care underscores the relevance of addressing cultural and communication needs in clinical practice [29]. In our study, nurses interpreted some apparent agreement with recommendations as potentially not reflecting full understanding or acceptance. This interpretation is consistent with the literature emphasizing the importance of avoiding assumptions about individual patients and considering structural and contextual factors in culturally responsive care [30].

Regarding the emotional impact of communication barriers, the nurses interviewed reported feelings of frustration and exhaustion. One participant (IDI-6) also described preferring not to work, when possible, in health centers with a high proportion of patients who did not speak Spanish. Rather than indicating burnout, the accounts suggest a considerable emotional and workload burden associated with repeated communication difficulties. Previous research has documented nurse burnout and its association with turnover and intention to leave the profession [31,32]. Participants in our study also described communication with migrant patients as requiring additional, often unrecognized effort, illustrated by the need to improvise drawings, pictograms, and gestures to facilitate explanations. Similar communication challenges have been reported in research on nurse–migrant patient encounters [24].

The accounts in our study also suggest that communication barriers do not affect all migrant patients equally and may be particularly challenging in some encounters with migrant women. Nurses perceived that limited social integration and reliance on partners could restrict some women’s ability to express their health needs autonomously during consultations. Previous research has reported gender- and origin-related differences in health care utilization among immigrant populations [33], while broader evidence identifies multiple interacting barriers and facilitators affecting migrants’ and refugees’ use of primary health care [34]. Thus, our findings point to a potential “meaning gap” that may extend beyond language proficiency alone. The literature on cultural competence in nursing education further emphasizes the importance of preparing nurses to recognize and respond to cultural diversity without relying on generalized assumptions about patients [35]. The description of some patients as “not as integrated” (IDI-17) may also reflect nurses’ own assumptions about social participation and language proficiency, rather than an objective assessment of patients’ integration. As these observations are based on nurses’ accounts, they reflect professional perceptions rather than the direct experiences of migrant women.

In the absence of formal institutional resources, nurses described relying on informal strategies, including drawings, gestures, digital translation tools, and mediation by third parties. These visual or digital tools may facilitate communication and help nurses address immediate clinical questions, although their effectiveness depends largely on the individual professional’s skills and resources [15]. Community social capital also emerged as an important source of support. Nurses highlighted the contribution of religious leaders, friends, and other patients who voluntarily provided translation assistance in waiting rooms. Although such community-based support can temporarily mitigate communication difficulties, reliance on voluntary and informal resources may reproduce inequities by making access to effective communication dependent on individual initiative and availability rather than on standardized institutional support [36].

Considering these findings, nurses identified organizational constraints, particularly insufficient training in intercultural care and limited institutional communication support, as major barriers. Cultural competence should be understood as an institutional responsibility rather than an individual professional attribute [37,38]. The lack of adequate resources and training may force nurses to rely on personal initiative to compensate for systemic deficiencies. This interpretation is consistent with the uncertainty expressed by IDI-20 about whether patients understood explanations and with IDI-10’s account of inaccurate digital translations, suggesting potential patient-safety implications rather than documented clinical errors. From an interpretive perspective, this reliance on individual improvisation can be understood as a form of professional survival in the face of recurring communication difficulties; the expression is used here to characterize the professional demands reflected in the accounts presented in the Results Section.

Finally, the nurses in this study proposed several concrete improvements, including standardized multilingual visual materials, access to professional interpreters, and real-time translation devices. Technological support may help reduce communication barriers and improve the perceived quality of care [12]. However, technological solutions should complement rather than replace professional training and institutional resources. Intercultural nursing education should therefore be formally incorporated into nursing curricula and continuing professional development [39]. More broadly, cultural competence should be systematically integrated into both nursing education and health care delivery to promote universal, equitable, and culturally responsive care [35,40].

### Limitations and Future Research Directions

Several limitations of this study should be acknowledged. First, the sample consisted exclusively of primary health care nurses from the province of Almería, which may limit the transferability of the findings to other geographical settings, health care systems, or levels of care. Second, the perspectives of migrant patients were not collected directly. Including their experiences would have allowed a more comprehensive understanding of communication barriers and comparison between nurses’ and patients’ perspectives. Finally, although in-depth interviews provided rich accounts of nurses’ experiences, direct observation of clinical practice could have complemented these accounts by capturing nonverbal communication and informal interactional practices that may not have been fully articulated during the interviews. In addition, access to potential participants was facilitated by primary care area coordinators, who hold managerial positions within the health care system. The coordinators helped identify eligible centers, contacted nurses, explained the study protocol, and invited voluntary participation; they did not conduct the interviews. Their involvement in facilitating recruitment may have influenced nurses’ decisions to participate or their perceived comfort when discussing institutional barriers and organizational practices. This potential influence should be considered when interpreting the findings, particularly those related to institutional resources and governance demands.

Considering the findings, several directions for future research can be identified. Rather than focusing solely on describing communication barriers, future studies should prioritize the development and evaluation of targeted interventions, including culturally and linguistically adapted digital health literacy programs and interventions addressing intersectional inequalities in nursing consultations. Further research should also examine how emerging technologies, including machine translation and artificial intelligence-based tools, may influence nurses’ experiences and contribute to safer and more effective communication. Finally, the effectiveness and real-world impact of the measures identified by participants should be evaluated, particularly their effects on quality and continuity of care, patient safety, and the satisfaction of nurses and migrant patients.

## 5. Conclusions

This study highlights primary care nurses’ perceptions of the communication barriers encountered when caring for migrant patients. Participants described recurrent misunderstandings and the need to repeat or adapt information, which they perceived as making treatment adherence, follow-up, vaccination, and continuity of care more challenging. Nurses also described an additional workload and emotional burden associated with these communication difficulties, including frustration, exhaustion, and concerns about potential misunderstandings in care.

Participants described communication difficulties as particularly challenging in some encounters with migrant women. According to nurses’ accounts, linguistic barriers could intersect with social isolation, dependence on partners or family members, and limited opportunities for women to express their health needs autonomously. Nurses also perceived that family dynamics and sociocultural beliefs could influence how health information and recommendations were understood and incorporated into everyday life. These findings highlight the need to consider linguistic, gender, and sociocultural dimensions alongside biomedical care.

When institutional resources were perceived as insufficient, nurses reported using informal nonverbal communication, pictograms, gestures, personal digital translation tools, and support from community members such as religious leaders, friends or other patients. Although participants described these strategies as useful for addressing immediate communication needs, they also identified limitations and risks associated with reliance on informal resources. From the perspective of the participating nurses, these findings support the need for institutional measures, including access to professional interpreting services, standardized multilingual materials, appropriate technological resources, and systematic training in culturally competent and intercultural care. At an organizational level, allowing adequate time for complex communication encounters may also be considered, although consultation time was not identified as a distinct finding in the interviews.

## Figures and Tables

**Table 1:** Sociodemographic characteristics of participants (N = 22).

Participant	Sex	Age (Years)	Professional Experience (Years)	Patient Populations Served by the Reported Country or Region of Origin
IDI-1	Male	37	2	Moroccan
IDI-2	Female	26	5	Moroccan
IDI-3	Female	43	17	Moroccan
IDI-4	Female	49	29	Moroccan
IDI-5	Female	34	8	British, German, Moroccan
IDI-6	Female	62	7	Moroccan
IDI-7	Female	49	25	Moroccan
IDI-8	Female	51	17	Moroccan and British
IDI-9	Female	53	11	Moroccan
IDI-10	Female	43	10	Moroccan, Romanian, Ukrainian
IDI-11	Male	52	27	Moroccan, Romanian, Ukrainian, other African origins, British, South American origins
IDI-12	Female	33	11	Moroccan, Romanian, Ukrainian, other African origins, British, South American origins
IDI-13	Female	37	16	Moroccan, Romanian, Ukrainian, other African origins, British, South American origins
IDI-14	Female	33	8	Moroccan, Romanian, Ukrainian, other African origins, British, South American origins
IDI-15	Female	36	14	Moroccan, Romanian, Ukrainian, other African origins, British, South American origins
IDI-16	Male	48	27	Moroccan, Romanian, Ukrainian, other African origins, British, South American origins
IDI-17	Female	37	14	Moroccan, Romanian, Ukrainian, other African origins, British, South American origins
IDI-18	Female	45	23	Moroccan, Romanian, Ukrainian, other African origins, British, South American origins
IDI-19	Female	47	23	Moroccan, Romanian, Ukrainian
IDI-20	Female	38	15	Moroccan
IDI-21	Male	47	23	Moroccan, Romanian, Ukrainian, other African origins, British, South American origins
IDI-22	Female	32	6	Moroccan and British

IDI = in-depth interview.

**Table 2:** Interview protocol.

Phase	Topic	Guiding Questions
Introduction	Purpose	To explore nurses’ experiences of communicating with migrant patients.
Introduction	Ethical considerations	Provide information about voluntary participation, audio-recording, informed consent, confidentiality, and the possibility of withdrawing from the study.
Beginning	Introductory question	Could you tell me about the migrant patient population served by your health center?
Development	Conversation guide	How would you describe your experience of communicating with migrant patients who do not speak Spanish? Could you share a situation in which a language barrier affected a patient’s diagnosis, treatment, or follow-up? How do you think language barriers affect patient’s perceptions of the care they receive? What strategies do you use to communicate with patients who do not share your language or culture background?
Closing	Final question	Is there anything else you would like to tell me?
Closing	Appreciation	Thank participants for their contribution, acknowledge the value of their participation, and offer them the opportunity to ask questions or raise any concerns.

**Table 3:** Example of coding strategy.

Quotation	Initial Code	Subtheme	Main Theme
“Many times, you are talking to them and they keep saying yes, yes, but in reality they do not understand what you are telling them” (IDI-7).	Inability to maintain fluent communication, recurrent misunderstandings.	3.1.1. Mutual misunderstanding in routine procedures	Theme 1. Language barriers, discontinuity of care, and nursing workload
“They are always at home; they do not go anywhere alone, not even to the health center. That means they hardly hear Spanish and everything depends on the husband” (IDI-13).	Domestic isolation and dependence on partners or third parties for health care interactions.	3.2.1. Gender determinants of linguistic isolation among women	Theme 2. Nurses’ perceptions of gender and cultural influences on communication

**Table 4:** Themes, subthemes, and meaning units.

Theme	Subtheme	Meaning Unit
Theme 1. Language barriers, discontinuity of care, and nursing workload	3.1.1. Mutual misunderstanding in routine procedures	Inability to maintain fluent communication, recurrent misunderstandings, repeated explanations, insecurity, and frustration.
	3.1.2. Treatment interruption and vaccination uncertainty	Treatment discontinuation, poor therapeutic adherence, difficulties with clinical follow-up, loss of continuity of care, and difficulties verifying childhood vaccination status.
	3.1.3. Emotional strain and self-protection strategies	Emotional and psychological burden among staff, lack of institutional support, frustration, physical and psychological exhaustion, fear of malpractice, professional self-protection, and professionals’ emotional and cultural self-awareness.
Theme 2. Nurses’ perceptions of gender and cultural influences on communication	3.2.1. Gender determinants of linguistic isolation among women	Gender disparities in language proficiency, limited social integration, domestic isolation, and dependence on partners or third parties for health care interactions.
	3.2.2. Sociocultural Determinants of Care: From the Family as a Cornerstone to the Impact of the Community Environment	Conceptions of care within the domestic and family sphere, differences in care relationships, nurses’ perceived differences between health centers.
Theme 3. Adaptive responses, community support, and governance demands	3.3.1. Nonverbal communication strategies and ad hoc technology	Improvised visual and nonverbal communication methods, pictographic charts, gestures, hand-drawn images, Google Translate, and risk of clinical errors.
	3.3.2. Social capital and spontaneous community mediation	Social capital, informal mediation, support from religious leaders, spontaneous assistance, and accompaniment by friends.
	3.3.3. Institutional gaps and proposals for improvement	Scarcity of official resources, guidelines, protocols, and formal training in cultural diversity; loss of telephone interpreting services; need for specific protocols, multilingual materials, translation devices, and organizational leadership.

## Data Availability

The data cannot be shared publicly due to their confidential nature. The anonymized qualitative data are available from the author [M.D.M.J.L.] upon reasonable request for academic or research purposes, as permitted by the institutional ethics approval and the participants’ informed consent. The original interview transcripts are in Spanish.

## Abbreviations

The following abbreviations are used in this manuscript:

COREQ	Consolidated Criteria for Reporting Qualitative Research
COVID-19	Coronavirus Disease 2019
EU	European Union
IDI	In-Depth Interview
UHC	Universal Health Coverage
WHO	World Health Organization
